# P-381. Enhancing Economic Outcomes by Strategic Investment in Orthopedic Surgical Infection Control

**DOI:** 10.1093/ofid/ofae631.582

**Published:** 2025-01-29

**Authors:** Raquel Bandeira, José Américo Bahia Filho, Vespasiano C Luz Neto, Glauco Messias, Gabrielle R Mota, Thiago C Gontijo, Gabriel Colen, Ana Carolina Morganti, Ana Paula Ladeira, Bráulio R G M Couto, Lívia Miranda, Isabela Messias, Beatriz Messias

**Affiliations:** Hospital Metropolitana Doutor Célio de Castro, Belo Horizonte, Minas Gerais, Brazil; Hospital Universitário Ciências Médicas (HUCM), Belo Horizonte, Minas Gerais, Brazil; Hospital Universitário Ciências Médicas (HUCM), Belo Horizonte, Minas Gerais, Brazil; Hospital Universitário Ciências Médicas (HUCM), Belo Horizonte, Minas Gerais, Brazil; Hospital Universitário Ciências Médicas (HUCM), Belo Horizonte, Minas Gerais, Brazil; University Hospital Medical Sciences, Belo Horizonte, Minas Gerais, Brazil; Hospital Universitário Ciências Médicas (HUCM), Belo Horizonte, Minas Gerais, Brazil; Hospital Universitário Ciências Médicas (HUCM), Belo Horizonte, Minas Gerais, Brazil; Biobyte Tecnologia em Epidemiologia, Belo Horizonte, Minas Gerais, Brazil; AMECI – Associação Mineira de Epidemiologia e Controle de Infecções, Belo Horizonte, Minas Gerais, Brazil; Faculdade Dinâmica Vale do Piranga - FADIP, Viçosa, Minas Gerais, Brazil; Hospital Universitário Ciências Médicas (HUCM), Belo Horizonte, Minas Gerais, Brazil; Hospital Universitário Ciências Médicas (HUCM), Belo Horizonte, Minas Gerais, Brazil

## Abstract

**Background:**

This study aims to assess the financial impact of investments in combating hospital-acquired infections, particularly surgical site infections (SSIs) in orthopedic procedures.

**Figure 1:**
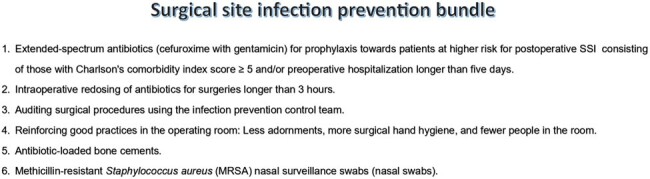
The infection prevention programs to prevent Surgical Site Infections (SSIs)

**Methods:**

A retrospective cohort study from January 2019 to September 2023 included patients undergoing knee arthroplasty (KA), hip arthroplasty (HA), and open fracture reduction (OFR), defining SSI according to CDC criteria. Costs per SSI patient were retrieved from literature sources: $13,539.50 for hospital costs (LUNA et al., 2022) and $7,523.77 for direct treatment costs, adjusted for inflation (STARLING et al., 2022).

**Figure 2.**
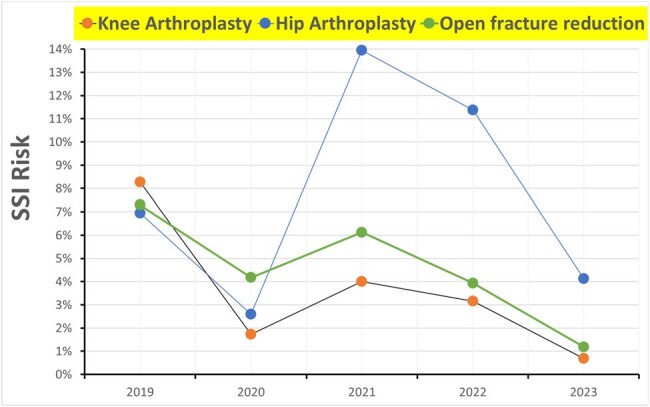
Chart of Year-to-Year Surgical Site Infection Rates, Stratified by Type of Orthopedic Surgery

Chart of Year-to-Year Surgical Site Infection Rates, Stratified by Type of Orthopedic Surgery

**Results:**

During the baseline period, January 2019 to to December 2022, 4,258 patients underwent orthopedic surgeries: HA (11%), KA (9%), OFR (80%). Of these, 2,439 were female (57%), 1,819 male (43%), mean and median age 51 years, standard deviation 21 years. 68 patients died in-hospital (1.6%), 239 diagnosed with SSI (5.6%). Postoperative infection increased mortality risk over twofold: without infection (1.5% mortality risk), with infection (3.8% mortality risk), relative risk 2.5, p-value=0.014. SSI was associated with prolonged hospitalization; average length of stay doubled with infection (18.2 days) compared to without (9 days), p-value: 0.001. Comparing infection risks from 2019-2022 (5.6%) to 2023 (1.4%) post-investment in infection control demonstrated a risk reduction. Relative risk (0.25) p-value < 0.001.

**Table 1.**
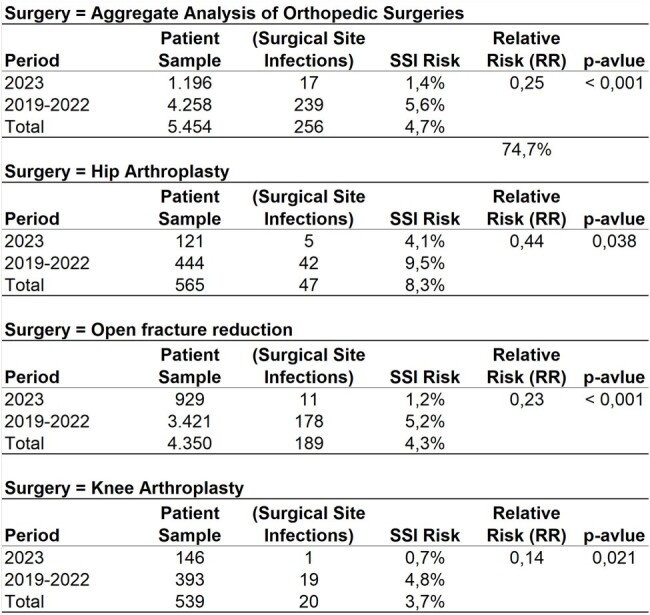
Annual Comparative Analysis of Surgical Site Infection Rates by Orthopedic Surgery Type

**Conclusion:**

SSIs in orthopedic surgeries pose dual risks, increasing readmissions and impairing hospital performance. Investing in SSI reduction enhances patient care, safety, and yields significant financial returns. This study underscores the ROI of infection prevention, especially targeting orthopedic surgical infections. Preventing SSIs can yield monthly savings of $25,781.49 to $46,393.48

**Table 2.**
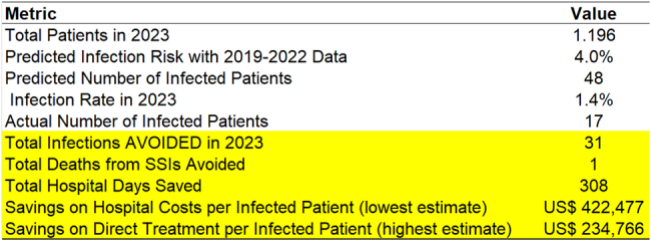
Reduction in Costs, Hospital Stay Duration, and Prevented Deaths Associated with Surgical Site Infections after Orthopedic Surgeries

**Disclosures:**

**All Authors**: No reported disclosures

